# Changes in Cholinesterase Activity in Blood of Adolescent with Metabolic Syndrome after Supplementation with Extract from* Aronia melanocarpa*

**DOI:** 10.1155/2018/5670145

**Published:** 2018-03-26

**Authors:** Piotr Duchnowicz, Anna Ziobro, Elżbieta Rapacka, Maria Koter-Michalak, Bożena Bukowska

**Affiliations:** ^1^Department of Biophysics of Environmental Pollution, Faculty of Biology and Environmental Protection, University of Łódź, Pomorska 141/143 St., 91–237 Łódź, Poland; ^2^Department of Normal and Clinical Anatomy, Interfaculty Chair of Anatomy and Histology, Medical University of Łódź, 60 Naturowicza St., 90–136 Łódź, Poland

## Abstract

Obesity and metabolic syndrome (MetS) are growing problems among children and adolescents. There are no reports of changes in the activity of butyrylcholinesterase (BChE) in children and adolescents with metabolic syndrome especially after supplementation with extract from* Aronia melanocarpa*. Materials studied included plasma and erythrocytes isolated from peripheral blood of patients with MetS and healthy subjects. We have estimated the following parameters: acetylcholinesterase (AChE) and butyrylcholinesterase (BChE) activity, lipid peroxidation and lipids levels in plasma, and erythrocytes membrane. In patients with MetS, a significant increase in AChE and BChE activity, higher LDL-cholesterol and triacylglycerol levels, and lower HDL-cholesterol level were observed. Supplementation with* A. melanocarpa* extract resulted in mild but statistically significant reduction of total cholesterol, LDL-cholesterol, and triacylglycerol levels and caused an increase in HDL-cholesterol level and a decrease in lipid peroxidation in plasma patients with MetS. Additionally, a decrease in lipid peroxidation and cholesterol level and a decrease in AChE activity in the erythrocyte membranes after supplementation with* A. melanocarpa* were noted. Summarizing, an increase in AChE and BChE activity and disruption of lipid metabolism in patients with MetS were observed. After supplementation of MetS patients with* A. melanocarpa* extract, a decrease in AChE activity and oxidative stress was noted.

## 1. Introduction

The metabolic syndrome (MetS) is defined as a group of risk factors of cardiovascular disease and type 2 diabetes mellitus, which include abdominal obesity, disorder of lipid metabolism, glucose intolerance, and hypertension. In 2005, the International Diabetes Federation (IDF) proposed aligning the definition of the MetS in adults. In 2007, the IDF published definitions of MetS in children and adolescents, which replaced absolute values of waist circumference with percentiles appropriate for age and sex [1].

The main causes of the development of the MetS are unbalanced diet, stress, reduced physical activity, and genetic factors. Easy access to high-energy food combined with low physical activity may lead to central obesity. Adipose tissue not only stores lipids but also releases a lot of adipocytokines [2]. Overproduction of adipocytokines may increase insulin resistance and blood pressure, causes oxidative stress, and disturbs lipid metabolism.

Treatment of MetS is difficult because of many factors influencing the development of this disease. The first stage of treatment is the change of diet and lifestyle. The increase in physical activity, low-calorie and low-fat diet, and increasing fruit and vegetables consumption cause weight loss that may lead to a significant reduction in the blood pressure and the improvement of lipid levels. Population studies have shown that the Mediterranean diet rich in fruits, vegetables, legumes, and grains is inversely associated with incidence of MetS [3].

An important role is attributed to polyphenolic compounds present in fruits and vegetables. Dietary polyphenols have strong antioxidant properties and can participate in the defense against oxidants. Antioxidant activity of polyphenols depends on the number and localization of the hydroxyl groups. Rice-Evans et al. [4] showed stronger antioxidant potential of flavonoids than vitamins C and E and carotenoids.* A. melanocarpa* (Michx.) Elliott. is one of the rich sources of procyanidins, anthocyanins, and phenolic acids. Oszmiański and Wojdylo [5] determined polyphenols, that is, procyanidins (about 66%), anthocyanins (about 25%), quercetin derivatives (about 1%), and chlorogenic and neochlorogenic acids (about 8%), as well as anthocyanins, that is, cyanidin 3-arabinose, cyanidin 3-galactoside, cyanidin 3-glucoside, and cyanidin 3-xyloside in chokeberry extract. Kulling and Rawel [6] conducted research on the antioxidant capacity of various fresh berries and fruits and preserves and showed that the fresh fruits and the juice of chokeberry had the highest antioxidant potential* in vitro*.

Oxidative stress defined as a change in the balance between an oxidative and an antioxidant system is associated with many diseases. Increased oxidative stress levels were observed in hypertension and ischemic disease [7], atherosclerosis [8], hypercholesterolemia [9], and diabetes [10]. A higher level of oxidative stress in adult patients with MetS was previously described [11]. Also, under physiological conditions, oxidants are produced, for example, as a result of increased metabolism associated with prolonged physical exercise [12]. In addition, oxidative stress intensifies with age as a result of the decrease in the efficiency of the antioxidant system. This may be partly due to the decrease in acetylcholinesterase activity in the erythrocyte membrane [13].

Cholinesterase belongs to a group of enzymes that hydrolyze acetylcholine and other choline esters. Human tissues have two distinct cholinesterase activities, that is, acetylcholinesterase (AChE) and butyrylcholinesterase (BChE). AChE is present in central nervous system and skeletal muscle and exists in the erythrocyte membranes, whereas BChE is secreted into plasma and synthesized in the liver. Erythrocytes AChE is located at the outside of the erythrocytes membrane [14] where it constitutes the Yt blood group antigen [15]. The physiological role of AChE is not known but has the same function as the enzyme involved in synaptic transmission, and its measurement is used to mirror the effects on the nervous system [16]. Although the function of human AChE located in the erythrocytes membrane is not well-known in these cells, the exact role of AChE bound with erythrocytes in the metabolic syndrome disease has been shown.

For a long time, the physiological function of BChE has been unclear. Patocka et al. [17] suggested that BChE participates in myelin maintenance, cellular adhesion, and neurogenesis and plays a role as a scavenger of toxic molecules.

So far, the studies concerning the activity of BChE in humans have included patients with hyperlipidemia, diabetes, or cardiovascular disease. Moreover, no research works on changes in BChE activity in children and adolescents with MetS have been conducted. Bearing that in mind, we have decided to estimate cholinesterase activities in blood of children with metabolic syndrome and compared these effects with changes in AChE and BChE activities in children supplemented with* A. melanocarpa* extract.

## 2. Materials and Methods

### 2.1. Study Population

The research was conducted on 77 nonsmoking patients aged 13–19. The children and adolescents (34 females and 32 males, age 13–19 years) who are referred to the Clinic of Endocrinology and Metabolic Diseases at Polish Mother's Memorial Research Institute and Medical University of Łódź, with a BMI ≥ 95th percentile for age and sex, were included in this study. Exclusion criteria were the presence of other associated chronic diseases, infections during the investigation period, four weeks preceding the study, and the use of any drugs.

During the entire study period patients had to maintain the current lifestyle without changes in diet or physical activity.

The reference group was 11 individuals (6 females and 5 males, age 14–18 years) from schools in Łódź, who agreed to participate in the study. The criterion for inclusion in the control group was correct body weight for age and sex. The exclusion from control group included chronic diseases, infections and inflammatory diseases during the investigation period, four weeks preceding the survey, and the use of any drugs.

All participants in our study had normal level of fasting plasma glucose (normal glucose tolerance). For subjects younger than 18 years, parents (or legal representative) agreed to participate in this study by signing the assent form. Subjects aged 18 years and older signed the informed consent form.

These experiments were conducted in accordance with ethical standards as formulated in the Helsinki Declaration of 1975 (revised 1983), consent numbers RNN/287/05/KB and KB/670/08/P of Commission of Medical Research Ethics of Medical University of Łódź, Poland.

### 2.2. Definition of Metabolic Syndrome

Modified criteria of the International Diabetes Federation (IDF) to diagnose MetS in children and adolescents: central obesity (waist circumference ≥ 90th percentile for age and sex) and any two of the following factors: raised triacylglycerol level ≥ 1.7 mmol/l, reduced HDL-cholesterol level ≤ 1.03 mmol/l, raised blood pressure (systolic ≥ 130 mm Hg and/or diastolic ≥ 85 mm Hg), and raised fasting plasma glucose ≥ 5.6 mmol/l or previously diagnosed type 2 diabetes [1].

### 2.3. Supplementation

Young people with MetS were treated with extract from* A. melanocarpa* (3 × 100 mg/day) for two months, without changing diet or lifestyle. The study used a commercial product under the name Aronox (Agropharm, Poland). According to the data from producer, 100 mg of Aronox (extract from fruits of* A. melanocarpa*) contains about 50 mg of polyphenols and no less than 20 mg of anthocyanins.

### 2.4. Serum and Erythrocytes Collection

Blood was collected on anticoagulant (23 mM citric acid, 45.1 mM sodium citrate, and 45 mM glucose) and then centrifuged for 600*g*  ×  10 min at 4°C to separate plasma and red blood cells. After centrifugation, serum was collected and analyzed for the selected parameters. The erythrocytes were washed three times with phosphate-buffered saline. The hematocrit level of the final erythrocyte suspensions was about 50%.

### 2.5. Butyryl and Acetylcholinesterase Activity

The activity of plasma butyrylcholinesterase (BChE; E.C. 3.1.1.8) and erythrocyte acetylcholinesterase (AChE; E.C. 3.1.1.7) was determined by the method of Ellman et al. [18]. We used specific substrates such as acetylthiocholine iodide (AcTCh) to estimate AChE activity and butyrylthiocholine iodide (BTC) to determine BChE activity.

One unit of AChE activity was defined as the amount of *μ*moles of acetylthiocholine degraded for 1 min by AChE contained in 1 mL of the erythrocytes of 100% hematocrit (packed cells).

One unit of BChE activity was defined as the amount of *μ*moles of butyrylthiocholine that was degraded for 1 min by BChE contained in 1 mL of the plasma.

### 2.6. Peroxidation of Lipids

Lipid peroxidation in plasma and in the erythrocytes was quantified by measuring the formation of thiobarbituric acid reactive substances (TBARS) [19]. The absorbance was read at 532 nm. The concentration of TBARS was calculated using a millimolar absorption coefficient for malonyl dialdehyde, *ε* = 1.56 × 10^5^ mol^−1^ dm^3^ cm^−1^. The results were expressed in *μ*mol MDA/L for plasma or *μ*mol MDA/g Hb for erythrocytes. The hemoglobin concentration was determined by Drabkin method [20]. Absorbance was read at 540 nm.

### 2.7. Cholesterol Concentration

The extraction of lipids from erythrocytes was carried out through the Rodríguez-Vico et al. method using chloroform/methanol mixture (2 : 1 v/v) [21]. The concentration of cholesterol was determined by Liebermann-Burchard reagent [22] and was expressed as milligrams of cholesterol per milliliter packed cells (mg HC/ml packed cells).

Serum total cholesterol (TC), HDL-cholesterol (HDL-C), LDL-cholesterol (LDL-C), and triacylglycerols (TG) were measured using enzymatic kits (ROCHE Diagnostic, Switzerland).

### 2.8. Total Plasma Antioxidant Capacity

The total antioxidant capacity (TAC) of the blood serum was estimated with the 2,2′-azonobis-(3-ethylbenzothiazoline-6-sulfonic acid) (ABTS^*∙*+^) absorbance capacity method [23]. Absorbance measurements at 734 nm were made after 10 s (rapidly reacting antioxidants, TAC “fast”) and after 30 min (long reacting antioxidants, TAC “slow”) of reaction time. The results were expressed in *μ*M of Trolox/L, using the relevant calibration curve.

### 2.9. Chemicals and Reagents

2-Thiobarbituric acid, Trolox, acetylthiocholine iodide, butyrylthiocholine iodide, and ABTS were purchased from Sigma-Aldrich (Poland). All other chemicals of product grade were purchased from POCh (Poland). Ultrapure water was produced in the laboratory using a Simplicity™ Water Purification System (Millipore, USA).

### 2.10. Statistical Analysis

The results are presented as mean ± SD. Differences between control and MetS patients groups were assessed by an unpaired Student's *t*-test. Differences between MetS patients before and after supplementation groups were assessed by a paired Student's *t*-test. The statistical analysis was performed using STATISTICA, version 10.0 PL software (StatSoft Inc., Tulsa, USA).

## 3. Results

Table 1 summarizes parameters for blood serum. Young patients with MetS had higher lipid peroxidation level (by 50%, *p* < 0.001), higher total cholesterol level (by 23%, *p* < 0.001), higher LDL-cholesterol level (by 29%, *p* < 0.005), higher triacylglycerol level (by 47%, *p* < 0.01), and lower HDL-cholesterol level (by 16%, *p* < 0.01) when compared to control group.

Lipid peroxidation decreased by 48% from baseline and reached control value after two months of supplementation with extract from chokeberry.

Two-month supplementation with chokeberry extract resulted in mild but statistically significant reduction in total cholesterol level by 4% and LDL-cholesterol level by 5% and an increase in HDL-cholesterol level by 3% comparing with the initial value, but these values were lower than value for the control group.

Triacylglycerol levels after two months of treatment with chokeberry extract decreased by 12% when compared to the initial value, but the value was still higher than that for the control.

No significant differences in TAC “fast” between control and MetS groups were noticed, while the value of TAC “slow” was lower in MS group when compared to control group (by 16%, *p* < 0.005). After two months of treatment with* A. melanocarpa* extract, TAC “fast” parameter increased by 5% from baseline and was significantly higher than that for the control by 8% (*p* < 0.05). TAC “fast” parameter increased by 9% from baseline and reached a control value.

**Figure 1:**
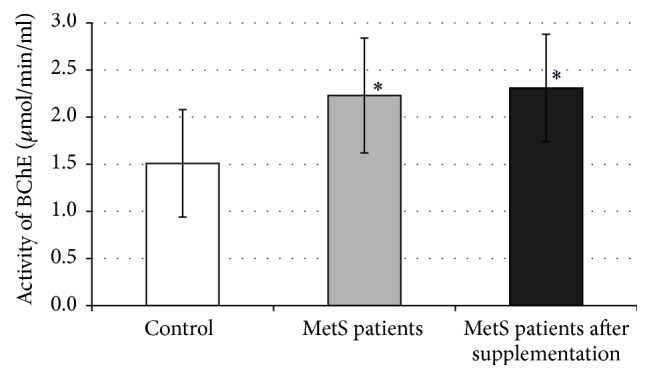
Activity of BChE in plasma. ^*∗*^*p* < 0.05 versus control.

BChE activity in patients with MetS was higher by 48% when compared to the control group (*p* < 0.001). After two months of supplementation with chokeberry extract, nonsignificant change in the enzyme activity was observed (Figure 1).

Table 2 summarizes parameters for the erythrocytes. Patients with MetS have a higher lipid peroxidation level and higher content of cholesterol in erythrocyte membranes when compared to the control by 28% and 42%, respectively. Lipid peroxidation in erythrocyte membranes decreased by 25% from baseline and reached a control value after two months of treatment with chokeberry extract. Also, the content of cholesterol in the erythrocyte membranes after treatment decreased by 6% when compared to the initial value but was still higher than control value.

**Figure 2:**
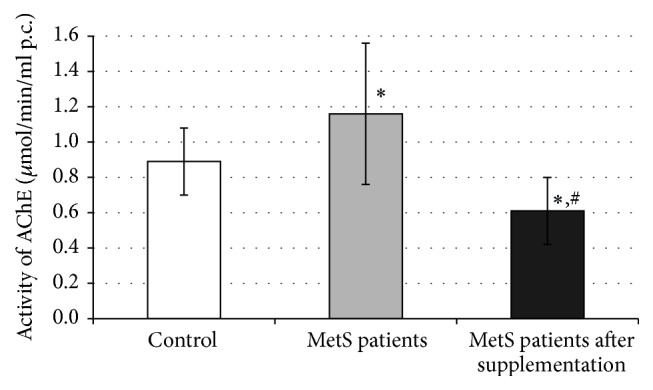
Activity of AChE in erythrocytes. ^*∗*^*p* < 0.05 versus control; ^#^*p* < 0.05 versus MetS patients.

AChE activity in patients with MetS was higher by 30% when compared to the control group (Figure 2). AChE activity decreased by 47% (*p* < 0.001) after two months of treatment with chokeberry extract when compared to baseline value and was lower by 31% (*p* < 0.05) than the activity of this enzyme in the control group.

## 4. Discussion

Published data suggest association between BChE activity and lipid metabolism and weight and body mass index (BMI) [24, 25]. Moreover, mean BChE activity tends to be higher in obese subjects than in nonobese individuals. Obese humans have high plasma BChE activity [24, 26], whereas starved humans have low plasma BChE activity [27].

Recently, researchers have suggested that one of the major functions of BChE is ghrelin hydrolysis, a unique octanoyl peptide that stimulates hunger and feeding. De Vriese et al. [28] showed that BChE cleaves of an acyl residue from ghrelin, which is a hormone involved in stimulation of growth hormone release, induction of adiposity, and weight gain by stimulating appetite. Tschöp et al. [29] observed reduced fasting plasma ghrelin levels in both adults and children. They proposed that decreased plasma ghrelin concentrations observed in obese patients represent a physiological adaptation to the positive energy balance associated with obesity.

Santarpia et al. [30] also suggested that, in obesity, metabolic syndrome, lipid metabolism impairment, and fatty liver, BChE activity can provide information on the patient's metabolism, habitual diet, and finally on the specific response to treatment.

In our study we have shown an increase in total blood cholesterol and cholesterol present in erythrocyte membranes as well as an increase in AChE and BChE activities in children with MetS when compared to control group. MetS patients had elevated LDL-cholesterol level (by about 29%) and triacylglycerol levels (by about 47%) while level of HDL-cholesterol was decreased by about 16%.

Many researchers have observed associations between the serum BChE activity and serum cholesterol and triacylglycerol levels in humans [26, 31]. The functional significance of serum BChE activity in individuals with risk factors for the metabolic syndrome is unclear but many research works have shown positive correlation between BChE activity and triacylglycerols, very low-density lipoprotein (VLDL), and LDL and a weak negative correlation between BChE activity and HDL level [32]. Lipoproteins have a phosphorylcholine group that can interact with BChE, which suggest that they may play a role in lipoprotein metabolism [33]. Also other scientists such as authors of [34, 35] showed that BChE plays a role in lipid metabolism, whether directly or through a synergistic action with cholesterol esterase.

Probably, high serum lipid concentrations may induce stereoscopic alteration in the enzyme configuration that modifies BChE activity or alters expression of enzyme-encoding gene, which determines BChE concentration and its activity [36].

Both cholinesterases are encoded by single genes, acetylcholinesterase by the AChE gene (chromosome 7q22.1), and butyrylcholinesterase by the BChE gene (chromosome 3q26.1-3q26.2). The UniProtKB database has 4 natural variants for AChE (P22303) and 44 natural variants for BChE (P06276) [https://www.uniprot.org/]. Lockridge compiled 75 natural variations of the human BChE gene described in 1987–2008. Most of these mutations are single mutations in regions located far away from the active site but causing loss of the butyrylcholinesterase activity [37]. The incidence of BChE variants* −116A* (in a noncoding region at exon 1) and* K* (at exon 4) was lower in obese than in nonobese adolescents, and compared to adult donors, which may suggest that these variants may protect adolescents from obesity [38]. In adults, the variant* −116A* occurs with the same frequency in obese and nonobese groups as opposed to variant* 1914G* (in a noncoding region at exon 4), the incidence of which is higher in obese individuals [39]. Regulation of gene expression can also take place using natural antisense transcripts that can act at different levels of transcription, RNA processing, and translation [40]. Additionally, microRNAs may be involved in the process of gene expression regulation. By combining in a specific way with mRNA they can cause both increase and decrease of protein expression [41, 42].

In patients with the CHE2 C− phenotype and with type I diabetes mellitus, lower BChE activity was determined compared to the CHE2 C5− phenotype and to type II diabetes mellitus. This difference was not observed among patients with the CHE2 C5+ phenotype. Also for patients with CHE2 C5− phenotype and obesity higher BChE activity was observed compared to nonobese patients [43].

Additionally, Alcântara et al. [44] proposed BChE to be secondary marker for metabolic syndrome in obese individuals with CHE2 C5− phenotype, while Rustemeijer et al. [35] suggested that BChE activity correlated with serum triacylglycerol and could therefore serve as a marker for the rate of triacylglycerol synthesis.

Stojanov et al. [45] demonstrated a direct association between the serum BChE activity and parameters of adiposity and lipids, that is, lipoprotein profile in young population. They proposed BMI as an independent risk factor for higher BChE activity in young men and glucose as an independent risk factor for higher BChE activity in women, which shows that changes in BChE activity may be a predictor of diabetes.

Adipose tissue is one of the sources of inflammatory cytokines such as tumor necrosis factor-alpha (TNF-*α*), interleukin-6 and interleukin-10 (IL-6, IL-10). Some authors demonstrated that obesity is associated with an inflammatory process, characterized by increased circulating levels of inflammatory cytokines such as TNF-*α*, IL-6, and C-reactive protein (CRP) [46, 47]. Recently, an increase has been demonstrated in the concentration of inflammatory markers in adolescents with MetS compared with non-MetS individuals. Additionally, in the group with MetS higher concentrations of TNF-*α*, IL-6, and CRP were noted for boys [48].

In our studies, the concentration of CRP was determined in control group and in group with MetS before supplementation. We also observed an increase in CRP in young patients with MetS compared with control group (mean 2.7 mg/l, range 0.5–9.7 mg/l versus mean 0.5 mg/l, range 0.1–1.0 mg/l, *p* < 0.001; date no shown).

It is known that high oxidative stress decreases BChE activity. Stefanello et al. [49] suggested that oxidative stress induced suppression of BChE activity probably via free radicals. Calderon-Margalit et al. [50] found that activity of BChE decreased significantly in subjects aged above 50 years and suggested a higher mortality risk for individuals with low BChE activity. Additionally, a decrease in plasma BChE activity was observed in hepatic diseases. Individuals with confirmed liver cirrhosis showed a 5-fold lower enzyme activity when compared to controls [51].

After two months of supplementation with chokeberry extract, we observed a significant decrease in lipid peroxidation and a slight decrease in total cholesterol content, while the activity of BChE remained unchanged and even slightly increased (by 4%). The observed decrease in oxidative stress did not affect the activity of BChE after supplementation with chokeberry extract. These results confirm that the activity of BChE is modulated to a greater extent by the level of cholesterol than by the level of oxidative stress.

It is also known that adult patients with MetS show higher levels of oxidative stress [11] and higher serum BChE activity [27]. In adults with MetS, two-month supplementation with extract from chokeberry resulted in a decrease in total cholesterol and LDL-cholesterol and a decrease in oxidative stress [52].

On the other hand, high oxidative stress and free radicals can increase AChE activity [53, 54]. Dal Forno et al. [55] suggested that an increment in ROS formation and a significant increase in lipid peroxidation could lead to the exposure of the more active sites of the AChE. The observed increase in AChE activity in basketball players after intensive training was caused by the increase in oxidation products, which was accompanied by increased metabolism [12].

The activity of AChE is also strongly modulated by the hydrophobic environment. Alcântara et al. [56] proposed hypothesis that cholesterol was incorporated into the cell membranes, which influenced the exposure of active site of AChE or affected the tertiary and quaternary structure of this enzyme.

Erythrocyte membranes from children with MS had elevated lipid peroxidation levels and cholesterol level and increased AChE activity. After two months of supplementation with chokeberry extract we observed a decrease in the level of lipid peroxidation and cholesterol content in erythrocytes membranes. Additionally, a decrease in the activity of AChE in patients with MetS supplemented with* A. melanocarpa* extract was observed.

The decrease in cholesterol and lipid peroxidation levels in the erythrocytes of children supplemented with* A. melanocarpa *extract caused a decrease in AChE activity.

Therefore, we noted that an increase in AChE and BChE activities appeared when lipid metabolism was abnormal, while a decrease in BChE activity and an increase in AChE activity occurred when oxidative stress was enhanced. The effect of high oxidative stress and impaired lipid metabolism on the increase in BChE activity was observed, which may play a significant role in lipid disorders.

## Figures and Tables

**Table 1:** Blood plasma parameters.

Parameters	Control group	MetS patients	MetS patients after supplementation	Control versus MetS patients	Before versus after supplementation
*N*	11	66	66		
Lipid peroxidation (*µ*mol MDA/l)	0.871 ± 0.185	1.304 ± 0.479	0.814 ± 0.241	0.001	0.05
Total cholesterol (mmol/l)	3.61 ± 0.30	4.44 ± 0.86	4.28 ± 0.83	0.001	0.05
LDL-cholesterol (mmol/l)	2.14 ± 0.31	2.76 ± 0.71	2.61 ± 0.73	0.005	0.05
HDL-cholesterol (mmol/l)	1.41 ± 0.31	1.18 ± 0.25	1.22 ± 0.24	0.01	0.05
Triacylglycerols (mmol/l)	0.86 ± 0.28	1.26 ± 0.60	1.11 ± 0.53	0.01	0.05
TAC “fast”: rapidly reacting antioxidants (*µ*M of Trolox/l)	515.6 ± 69.8	531.5 ± 82.6	558.1 ± 61.7	NS	NS
TAC “slow”: long reacting antioxidants (*µ*M of Trolox/l)	538.4 ± 79.1	452.2 ± 95.2	495.3 ± 70.2	0.01	0.01

**Table 2:** Erythrocytes membrane parameters.

Parameters	Control group	MetS patients	MetS patients after supplementation	Control versus MetS patients	Before versus after supplementation
*N*	11	66	66		
Lipid peroxidation (nmol MDA/g Hb)	30.3 ± 0.85	38.7 ± 1.24	29.2 ± 0.68	0.05	0.001
Cholesterol (mg/ml_p.c._)	2.57 ± 0.72	3.64 ± 0.60	3.43 ± 0.60	0.001	0.05

## References

[1] Zimmet P., Alberti G., Kaufman F. (2007). The metabolic syndrome in children and adolescents. *The Lancet*.

[2] Lau D. C. W., Dhillon B., Yan H., Szmitko P. E., Verma S. (2005). Adipokines: molecular links between obesity and atheroslcerosis. *American Journal of Physiology-Heart and Circulatory Physiology*.

[3] Tortosa A., Bes-Rastrollo M., Sanchez-Villegas A., Basterra-Gortari F. J., Nũnez-Cordoba J. M., Martinez-Gonzalez M. A. (2007). Mediterranean diet inversely associated with the incidence of metabolic syndrome: The SUN prospective cohort. *Diabetes Care*.

[4] Rice-Evans C. A., Miller N. J., Paganga G. (1997). Antioxidant properties of phenolic compounds. *Trends in Plant Science*.

[5] Oszmiański J., Wojdylo A. (2005). Aronia melanocarpa phenolics and their antioxidant activity. *European Food Research and Technology*.

[6] Kulling S. E., Rawel H. M. (2008). Chokeberry (Aronia melanocarpa)—a review on the characteristic components and potential health effects. *Planta Medica*.

[7] Correa M. d. C., Maldonado P., da Rosa C. S. (2008). Oxidative stress and erythrocyte acetylcholinesterase (AChE) in hypertensive and ischemic patients of both acute and chronic stages. *Biomedicine & Pharmacotherapy*.

[8] Roberts C. K., Barnard R. J., Sindhu R. K., Jurczak M., Ehdaie A., Vaziri N. D. (2006). Oxidative stress and dysregulation of NAD(P)H oxidase and antioxidant enzymes in diet-induced metabolic syndrome. *Metabolism*.

[9] Koter M., Franiak I., Strychalska K., Broncel M., Chojnowska-Jezierska J. (2004). Damage to the structure of erythrocyte plasma membranes in patients with type-2 hypercholesterolemia. *The International Journal of Biochemistry & Cell Biology*.

[10] Ramakrishna V., Jailkhani R. (2008). Oxidative stress in non-insulin-dependent diabetes mellitus (NIDDM) patients. *Acta Diabetologica*.

[11] Ziobro A., Duchnowicz P., Mulik A., Koter-Michalak M., Broncel M. (2013). Oxidative damages in erythrocytes of patients with metabolic syndrome. *Molecular and Cellular Biochemistry*.

[12] Parthimos T., Tsopanakis C., Angelogianni P., Schulpis K. H., Parthimos N., Tsakiris S. (2007). The effect of basketball training on the players' erythrocyte membrane acetylcholinesterase, (Na+,K+)-ATPase and Mg 2+-ATPase activities. *International Journal of Sports Medicine*.

[13] Jha R., Rizvi S. I. (2009). Age-dependent decline in erythrocyte acetylcholinesterase activity: correlation with oxidative stress. *Biomedical Papers*.

[14] Igisu H., Matsumura H., Matsuoka M. (1994). Acetylcholinesterase in the erythrocyte membrane. *Journal of UOEH*.

[15] Čolović M. B., Krstić D. Z., Lazarević-Pašti T. D., Bondžić A. M., Vasić V. M. (2013). Acetylcholinesterase inhibitors: pharmacology and toxicology. *Current Neuropharmacology*.

[16] Lotti M. (1995). Cholinesterase inhibition: Complexities in interpretation. *Clinical Chemistry*.

[17] Patocka J., Kuca K., Jun D. (2004). Acetylcholinesterase and butyrylcholinesterase--important enzymes of human body.. *Acta medica (Hradec Králové) / Universitas Carolina, Facultas Medica Hradec Králové*.

[18] Ellman G. L., Courtney K. D., Andres V., Featherstone R. M. (1961). A new and rapid colorimetric determination of acetylcholinesterase activity. *Biochemical Pharmacology*.

[19] Stocks J., Dormandy T. L. (1971). The autoxidation of human red cell lipids induced by hydrogen peroxide. *British Journal of Haematology*.

[20] Drabkin D. L. (1946). Spectrophotometric studies; the crystallographic and optical properties of the hemoglobin of man in comparison with those of other species. *The Journal of Biological Chemistry*.

[21] Rodríguez-Vico F., Martínez-Cayuela M., Zafra M. F., Garcia-Peregrin E., Ramírez H. (1991). A procedure for the simultaneous determination of lipid and protein in biomembranes and other biological samples. *Lipids*.

[22] Kim E., Goldberg M. (1969). Serum cholesterol assay using a stable Liebermann-Burchard reagent.. *Clinical Chemistry*.

[23] Janaszewska A., Bartosz G. (2009). Assay of total antioxidant capacity: comparison of four methods as applied to human blood plasma. *Scandinavian Journal of Clinical & Laboratory Investigation*.

[24] Furtado-Alle L., Andrade F. A., Nunes K., Mikami L. R., Souza R. L. R., Chautard-Freire-Maia E. A. (2008). Association of variants of the -116 site of the butyrylcholinesterase BCHE gene to enzyme activity and body mass index. *Chemico-Biological Interactions*.

[25] Li B., Duysen E. G., Lockridge O. (2008). The butyrylcholinesterase knockout mouse is obese on a high-fat diet. *Chemico-Biological Interactions*.

[26] Randell E. W., Mathews M. S., Zhang H., Seraj J. S., Sun G. (2005). Relationship between serum butyrylcholinesterase and the metabolic syndrome. *Clinical Biochemistry*.

[27] Waterlow J. C. (1970). Enzyme changes in malnutrition. *Journal of Clinical Pathology*.

[28] De Vriese C., Gregoire F., Lema-Kisoka R., Waelbroeck M., Robberecht P., Delporte C. (2004). Ghrelin degradation by serum and tissue homogenates: identification of the cleavage sites. *Endocrinology*.

[29] Tschöp M., Weyer C., Tataranni P. A., Devanarayan V., Ravussin E., Heiman M. L. (2001). Circulating ghrelin levels are decreased in human obesity. *Diabetes*.

[30] Santarpia L., Grandone I., Contaldo F., Pasanisi F. (2013). Butyrylcholinesterase as a prognostic marker: a review of the literature. *Journal of Cachexia, Sarcopenia and Muscle*.

[31] Sato K. K., Hayashi T., Maeda I. (2014). Serum butyrylcholinesterase and the risk of future type 2 diabetes: The Kansai Healthcare Study. *Clinical Endocrinology*.

[32] Cucuianu M., Popescu T. A., Opincaru A., Hǎrǎgus S. (1975). Serum pseudocholinesterase and ceruloplasmin in various types of hyperlipoproteinemia. *Clinica Chimica Acta*.

[33] Kutty K. M., Payne R. H. (1994). Serum pseudocholinesterase and very‐low‐density lipoprotein metabolism. *Journal of Clinical Laboratory Analysis*.

[34] Finer Y., Jaffer F., Santerre J. P. (2004). Mutual influence of cholesterol esterase and pseudocholinesterase on the biodegradation of dental composites. *Biomaterials*.

[35] Rustemeijer C., Schouten J. A., Voerman H. J., Beynen A. C., Donker A. J. M., Heine R. J. (2001). Is pseudocholinesterase activity related to markers of triacylglycerol synthesis in type II diabetes mellitus?. *Clinical Science*.

[36] Kálmán J., Juhász A., Rakonczay Z. (2004). Increased serum butyrylcholinesterase activity in type IIb hyperlipidaemic patients. *Life Sciences*.

[37] Lockridge O. (2015). Review of human butyrylcholinesterase structure, function, genetic variants, history of use in the clinic, and potential therapeutic uses. *Pharmacology & Therapeutics*.

[38] Chaves T. J., Leite N., Milano G. E. (2013). -116A and K BCHE gene variants associated with obesity and hypertriglyceridemia in adolescents from Southern Brazil. *Chemico-Biological Interactions*.

[39] Lima J. K., Leite N., Turek L. V. (2013). 1914G variant of BCHE gene associated with enzyme activity, obesity and triglyceride levels. *Gene*.

[40] Xi Q., Gao N., Zhang X. (2014). A natural antisense transcript regulates acetylcholinesterase gene expression via epigenetic modification in Hepatocellular Carcinoma. *The International Journal of Biochemistry & Cell Biology*.

[41] Hanin G., Shenhar-Tsarfaty S., Yayon N. (2014). Competing targets of microRNA-608 affect anxiety and hypertension. *Human Molecular Genetics*.

[42] Shaked I., Meerson A., Wolf Y. (2009). MicroRNA-132 potentiates cholinergic anti-inflammatory signaling by targeting acetylcholinesterase. *Immunity*.

[43] Cwiertnia M. M., Alcântara V. M., Réa R. R. (2010). Butyrylcholinesterase and diabetes mellitus in the CHE2 C5- and CHE2 C5+ phenotypes. *Arquivos Brasileiros de Endocrinologia & Metabologia*.

[44] Alcântara V. M., Oliveira L. C., Réa R. R., Suplicy H. L., Chautard-Freire-Maia E. A. (2005). Butyrylcholinesterase activity and metabolic syndrome in obese patients. *Clinical Chemistry and Laboratory Medicine*.

[45] Stojanov M., Stefanović A., Džingalašević G., Mandić-Radić S., Prostran M. (2011). Butyrylcholinesterase activity in young men and women: Association with cardiovascular risk factors. *Clinical Biochemistry*.

[46] Rodríguez-Hernández H., Simental-Mendía L. E., Rodríguez-Ramírez G., Reyes-Romero M. A. (2013). Obesity and inflammation: epidemiology, risk factors, and markers of inflammation. *International Journal of Endocrinology*.

[47] Sinicato N. A., Postal M., Peres F. A. (2014). Obesity and cytokines in childhood-onset systemic lupus erythematosus. *Journal of Immunology Research*.

[48] González-Jiménez E., Schmidt-Riovalle J., Sinausía L., Carmen Valenza M., Perona J. S. (2016). Predictive value of ceruloplasmin for metabolic syndrome in adolescents. *BioFactors*.

[49] Stefanello F. M., Franzon R., Tagliari B., Wannmacher C. M. D., Wajner M., Wyse A. T. S. (2005). Reduction of butyrylcholinesterase activity in rat serum subjected to hyperhomocysteinemia. *Metabolic Brain Disease*.

[50] Calderon-Margalit R., Adler B., Abramson J. H., Gofin J., Kark J. D. (2006). Butyrylcholinesterase activity, cardiovascular risk factors, and mortality in middle-aged and elderly men and women in Jerusalem. *Clinical Chemistry*.

[51] Ramachandran J., Sajith K. G., Priya S., Dutta A. K., Balasubramanian K. A. (2014). Serum cholinesterase is an excellent biomarker of liver cirrhosis.. *Tropical gastroenterology : official journal of the Digestive Diseases Foundation*.

[52] Broncel M., Koziróg M., Duchnowicz P., Koter-Michalak M., Sikora J., Chojnowska-Jezierska J. (2010). Aronia melanocarpa extract reduces blood pressure, serum endothelin, lipid, and oxidative stress marker levels in patients with metabolic syndrome. *Medical Science Monitor*.

[53] Bukowska B., Sicińska P., Pajak A. (2015). Oxidative stress and damage to erythrocytes in patients with chronic obstructive pulmonary disease - changes in ATPase and acetylcholinesterase activity. *The International Journal of Biochemistry & Cell Biology*.

[54] Yamchuen P., Aimjongjun S., Limpeanchob N. (2014). Oxidized low density lipoprotein increases acetylcholinesterase activity correlating with reactive oxygen species production. *Neurochemistry International*.

[55] Dal Forno G. O., Kist L. W., De Azevedo M. B. (2013). Intraperitoneal exposure to nano/microparticles of fullerene (C_60_) increases acetylcholinesterase activity and lipid peroxidation in adult zebrafish (danio rerio) Brain. *BioMed Research International*.

[56] Alcântara V. M., Chautard-Freire-Maia E. A., Scartezini M., Cerci M. S. J., Braun-Prado K., Picheth G. (2002). Butyrylcholinesterase activity and risk factors for coronary artery disease. *Scandinavian Journal of Clinical & Laboratory Investigation*.

